# Analysis of carries in signed digit expansions

**DOI:** 10.1007/s00605-016-0917-x

**Published:** 2016-06-10

**Authors:** Clemens Heuberger, Sara Kropf, Helmut Prodinger

**Affiliations:** 1 Institut für Mathematik, Alpen-Adria-Universität Klagenfurt, Universitätsstraße 65–67, 9020 Klagenfurt, Austria; 2 Department of Mathematical Sciences, Stellenbosch University, 7602 Stellenbosch, South Africa

**Keywords:** Carry, Central limit theorem, Transducer, Probabilistic automaton, Symmetric signed digit expansion, Von Neumann’s addition, 11A63, 60C05, 60F05, 68Q45, 68W40

## Abstract

The number of positive and negative carries in the addition of two independent random signed digit expansions of given length is analyzed asymptotically for the (*q*, *d*)-system and the symmetric signed digit expansion. The results include expectation, variance, covariance between the positive and negative carries and a central limit theorem. Dependencies between the digits require determining suitable transition probabilities to obtain equidistribution on all expansions of given length. A general procedure is described to obtain such transition probabilities for arbitrary regular languages. The number of iterations in von Neumann’s parallel addition method for the symmetric signed digit expansion is also analyzed, again including expectation, variance and convergence to a double exponential limiting distribution. This analysis is carried out in a general framework for sequences of generating functions.

## Introduction

Addition is an essential arithmetic operation in many algorithms. As the efficiency of addition is influenced by the number of occurring carries, we asymptotically analyze this number, which depends on the base and the digit set of the digit expansion.

We consider two different types of digit expansions: On the one hand, we investigate (*q*, *d*)-expansions, that are extensions of the standard *q*-ary digit expansion with digit set $\{d,\ldots ,q+d-1\}$. With $d=0$, this includes the case of the standard *q*-ary expansion. Consecutive digits are independent in this case. On the other hand, the symmetric signed digit expansion [6] has an even base *q* and the redundant digit set $\{-q/2,\ldots ,q/2\}$. To remove the redundancy, there is a syntactical rule to decide which of the digits $-q/2$ and *q* / 2 is used. This rule introduces dependencies between consecutive digits.

Two different addition algorithms are investigated. The first one is the standard addition: We add two digits starting at the least significant position. If the result is not in the given digit set or does not fulfill the syntactical conditions, then a non-zero carry is produced. This carry is added to the sum of the two digits at the next position. An example for this standard addition of two decimal expansions is given in Table 1.

**Table 1:**
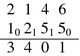
Example for standard addition in the decimal system

The subscripts in the second row are the carries

In the case of positive and negative digits, positive and negative carries occur. The parameter of interest is their number for an independent pair of random summands of given length.

In contrast to standard addition, von Neumann’s addition is a parallel algorithm with several iterations. The idea is to add the digits at each position in parallel (the interim result). If this result is not admissible in the given digit system, then a non-zero carry is produced and the interim result is corrected correspondingly at this position. However, this carry is not added immediately: The interim result and the carries are the input for the next iteration. When the carry sequence only contains zeros, then the algorithm terminates. An example for von Neumann’s addition is shown in Table 2 for the addition of two decimal expansions.

**Table 2:**
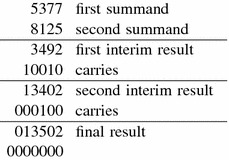
Example for von Neumann’s addition in the decimal system

The number of iterations of von Neumann’s addition is of interest as it corresponds to the running time.

Diaconis and Fulman [1] and Nakano and Sadahiro [9] consider the carries of the standard addition as a Markov chain. This is only valid if the digits of the digit expansion are independent. In their analysis, they obtain a stationary distribution. In this article, we determine the expectation, variance and central limit theorem for the number of positive and negative carries as well as the covariance between the positive and negative carries in the (*q*, *d*)-system and the symmetric signed digit system. The authors of [1] concentrate on an *odd* basis *q* and the symmetric digit set $\{-(q-1)/2,\ldots ,(q-1)/2\}$. The symmetric signed digit expansion (defined later) is the natural way to define a unique representation with a symmetric set of digits and an *even* base *q*. Thus, a part of the present paper can be seen as a complement of [1].

The expected number of iterations of von Neumann’s addition was analyzed in [8] and [7] for standard *q*-ary expansions and (*q*, *d*)-expansions, respectively. It turns out that the expected number of iterations is logarithmic in the length of the expansions. In [7], symmetric signed digit expansions are analyzed, too, but with a simplified probabilistic model since a precise probabilistic model exceeded computing resources available at that time. This simplification has a significant influence on the main term. In this paper, we combine advances in soft- and hardware with sophisticated use of the finite state machine package of SageMath [13] to tackle the precise model in roughly 10 minutes of CPU time. The results include expectation, variance and convergence to a double exponential distribution.

The outline of the paper is as follows. In Sect. 2, we define (*q*, *d*)-expansions and symmetric signed digit expansions. We first analyze the standard addition in Sects. 3–5. The algorithms and the corresponding transducers for the standard addition of (*q*, *d*)-expansions and symmetric signed digit expansions are presented in Sect. 3. Our probabilistic model is to choose both summands of length $\ell$ independently such that each expansion of length $\ell$ is equally likely. In the case of the symmetric signed digit expansions, the dependencies between the digits require approximating the equidistribution with an error that does not influence the final result. The corresponding probabilities are defined in Lemma 4.1 in Sect. 4 for general regular languages, see also [10, 14]. In Sect. 5, we combine this approximate equidistribution with the transducers from Sect. 3.2 to obtain an asymptotic analysis including the expectation, the variance and asymptotic normality in the main Theorems 1 and 2 for the (*q*, *d*)-system and the symmetric signed digit system, respectively.

Then, we analyze von Neumann’s addition. We start in Sect. 6 with the algorithms and the automaton. Theorem 3 provides a general framework for the analysis of sequences occurring in this context. Then we again use the approximate equidistribution from Sect. 4 to asymptotically analyze the number of iterations of von Neumann’s addition in Theorem 4 in Sect. 7. This analysis extends the results in [7, 8] to the symmetric signed digit expansions and to include not only the expected value but also the variance and a convergence in distribution.

Obtaining the values of the constants occuring in the asymptotic analysis of standard and von Neumann’s addition requires computations involving finite state machines and determinants of matrices in several variables. These computations are performed using the mathematical software system SageMath [13]. Notebooks containing all the computations can be found at [4]. However, the existence of these constants follows from the theoretical results.

## Digit expansions

In this section, we define the digit expansions which will be used in later sections. We also recall their properties.

### (*q*, *d*)-expansions

#### Definition 2.1

Let $-q<d\le 0$ be two integers with $q\ge 2$. The (*q*, *d*)-expansion of an integer *x* is the *q*-ary expansion $(x_{\ell }\ldots x_{0})_{q}$ with digits $x_{i}\in \{d,\ldots , q+d-1\}$ such that $x=\sum _{i=0}^{\ell }x_{i}q^{i}$.

#### Example 2.2

The $(4,-1)$-expansion of 3 is $(1\bar{1})_{4}$, where we write $\bar{1}$ for the digit $-1$.

The (*q*, *d*)-expansion exists for all integers if $d\ne 0$ and $d\ne -q+1$. For $d=0$ (this is the standard *q*-ary expansion), only the non-negative integers have a (*q*, *d*)-expansion. Conversely, for $d=-q+1$, only the non-positive integers have a (*q*, *d*)-expansion. If the (*q*, *d*)-expansion of an integer exists, then it is unique up to leading zeros.

If *q* is odd and $d=\frac{-q+1}{2}$, then the (*q*, *d*)-expansion minimizes the sum of absolute values of the digits among all *q*-ary expansions with arbitrary digits (see [6]).

### Symmetric signed digit expansion

We recall the definition of the symmetric signed digit expansion (SSDE) as defined in [6] and further analyzed in [7].

#### Definition 2.3

Let $q\ge 2$ be an even integer. The symmetric signed digit expansion (SSDE) of an integer is the *q*-ary digit expansion $(x_{\ell }\ldots x_{0})_{q}$ with $x_{i}\in \{-\frac{q}{2},\ldots ,\frac{q}{2}\}$ such that the syntactical rule

$$\begin{aligned} |x_{j}|=\frac{q}{2}\quad \Longrightarrow \quad 0\le {{\mathrm{sgn}}}(x_{j})x_{j+1}\le \frac{q}{2}-1 \end{aligned}$$

is satisfied for $0\le j<\ell$.

In [6], it is shown that each integer *n* has a unique SSDE (up to leading zeros). It minimizes the sum of absolute values of the digits among all *q*-ary expansions of *n* with arbitrary digits (cf. [6]).

For $q=2$, we obtain the digit set $\{0,\pm 1\}$ and the syntactical rule that at least one of any two adjacent digits is zero. This digit expansion is also called non-adjacent form (cf. [12]).

## Standard addition

We write bold face letters for sequences which are padded with zeros on the left.

Let $\varvec{x}=\ldots x_{1}x_{0}$ and $\varvec{y}=\ldots y_{1}y_{0}$ be the two summands given as *q*-ary expansions with digit set *D* (possibly satisfying some syntactical rules). Then standard addition can be written in the formwhere $x_{i}+y_{i}+c_{i}=z_{i}-qc_{i+1}$, $c_{0}=0$ with $z_{i}\in D$ and $\varvec{z}=\ldots z_{1}z_{0}$ satisfying the syntactical rules of the digit system under consideration. We asymptotically analyze the sequence of carries $\varvec{c}=\ldots c_{2}c_{1}$.

From a different point of view, the standard addition with digit set *D* is a conversion between different digit sets: We have a *q*-ary digit expansion with digits in $D+D$ and we want to transform this digit expansion into a digit expansion with digit set *D* satisfying all syntactical rules. This can be written in the formwhere $s_{i}=x_{i}+y_{i}\in D+D$. We call the sequence $\varvec{s}$ the digitwise sum of $\varvec{x}$ and $\varvec{y}$ and write $\varvec{s}=\varvec{x}+\varvec{y}$.

We will mostly use this point of view. Most of the algorithms and transducers require the input of $\varvec{s}$. If there are syntactical rules for $\varvec{x}$ and $\varvec{y}$, then the sequence $\varvec{s}$ can not be arbitrary.

### Remark 3.1

From this point of view, it is clear that interchanging two digits $x_{i}$ and $y_{i}$ of the two summands does not influence the result, but only both summands. The carries, the digitwise sum and the steps taken by the algorithms and the transducers stay the same as they depend only on the digitwise sum.

### Algorithms

#### Standard addition for (*q*, *d*)-expansions

The digit set is $D=\{d,\ldots , q+d-1\}$. Algorithm 1 transforms a *q*-ary expansion with digit set $D+D$ into a (*q*, *d*)-expansion. As there are no syntactical rules, all digits are independent. Thus, we do not have to look ahead when choosing the carry.

An example of standard addition for $(5,-1)$-expansions using this algorithm is given in Table 3.

**Table 3:**
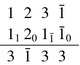
Example for standard addition for $(5,-1)$-expansions

The subscripts in the second row are the carries


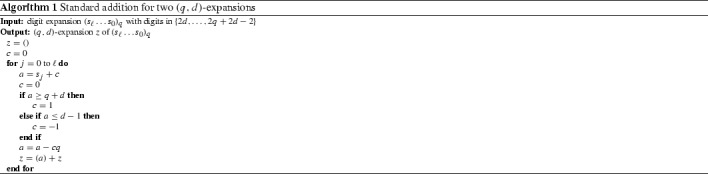


#### Standard addition for SSDEs

Let $q\ge 2$ be even. Algorithm 2 transforms a *q*-ary expansion with digit set $\{-q,\ldots ,q\}$ into a SSDE. As the choice between the redundant digits $\frac{q}{2}$ and $-\frac{q}{2}$ depends on the next digit, we have to look ahead at the next digit in these cases. This algorithm is an extension of the one in [7] taking into account that we start with a larger digit set.

An example of standard addition for SSDEs with $q=4$ using this algorithm is given in Table 4.

**Table 4:**
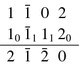
Example for standard addition for SSDEs for $q=4$

The subscripts in the second row are the carries


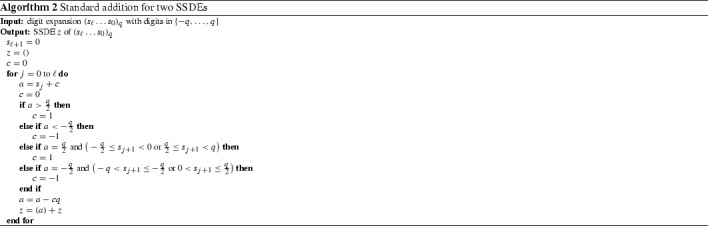


### Transducers

In this section, we present the transducers for the algorithms presented in the last section.

We are not interested in the output of the addition, but only in the carries. Thus we only use the carries as the output of the transducer. But, if required, the output digits can easily be reconstructed.

In our setting, a transducer consists of a finite set of states *S*, a finite input alphabet $D+D$, an output alphabet, a set of transitions $E\subseteq S^{2}\times (D+D)$ with input labels in $D+D$, output labels in the output alphabet for each transition, and an initial state. All states are final.

The input of the transducer is a digit expansion with digits in $D+D$. The output of the transducer is the sequence of labels of a path starting in the initial state with the given input as the input label. In our cases, always exists such a path and it is unique (i.e., the transducer is complete and deterministic).

The labels of the states encode the current carry (except for the situations when we have to look ahead). The number of states is independent of *q*. The number of transitions between two states depends on the base *q*.

To plot the transducer, we group these transitions and their labels. We draw only one arc and write the label $M\mid c$ for a set $M\subset D+D$ to represent a group of transitions consisting of one transition with input label *m* and output label *c* for every $m\in M$. If *M* is the empty set, then there are no such transitions. This may happen for special values of *d* or *q*.

The output label of a transition is one carry *c*, a pair of carries *c*, or no carry *c*, i.e., $c\in \{0,1,\bar{1},-\}\cup \{0,1,\bar{1}\}^{2}$, where “−” denotes the empty output. The input of the transducer is the sequence $\varvec{s}$ of digitwise sums.

Let $\ell$ and *u* be the minimum and the maximum of the extended digit set $D+D$. For the labels of the transitions, we define

$$\begin{aligned} M+\varepsilon&=\big (\{m+\varepsilon \mid m\in M\}\cup (M\cap \{\ell ,u\})\big )\cap (D+D) \end{aligned}$$

for $\varepsilon =\pm 1$ and a set *M*. This definition is motivated by the following interpretation: Whenever a set $M=\{j, \ldots , u\}$ occurs, it is actually meant to be the interval $[j, \infty )$ intersected with the extended digit set. Subtracting 1 leads to $[j-1, \infty )$, again intersected with the extended digit set. This corresponds to $M-1$ as defined above.

#### Standard addition for (*q*, *d*)-expansions

**Fig. 1:**
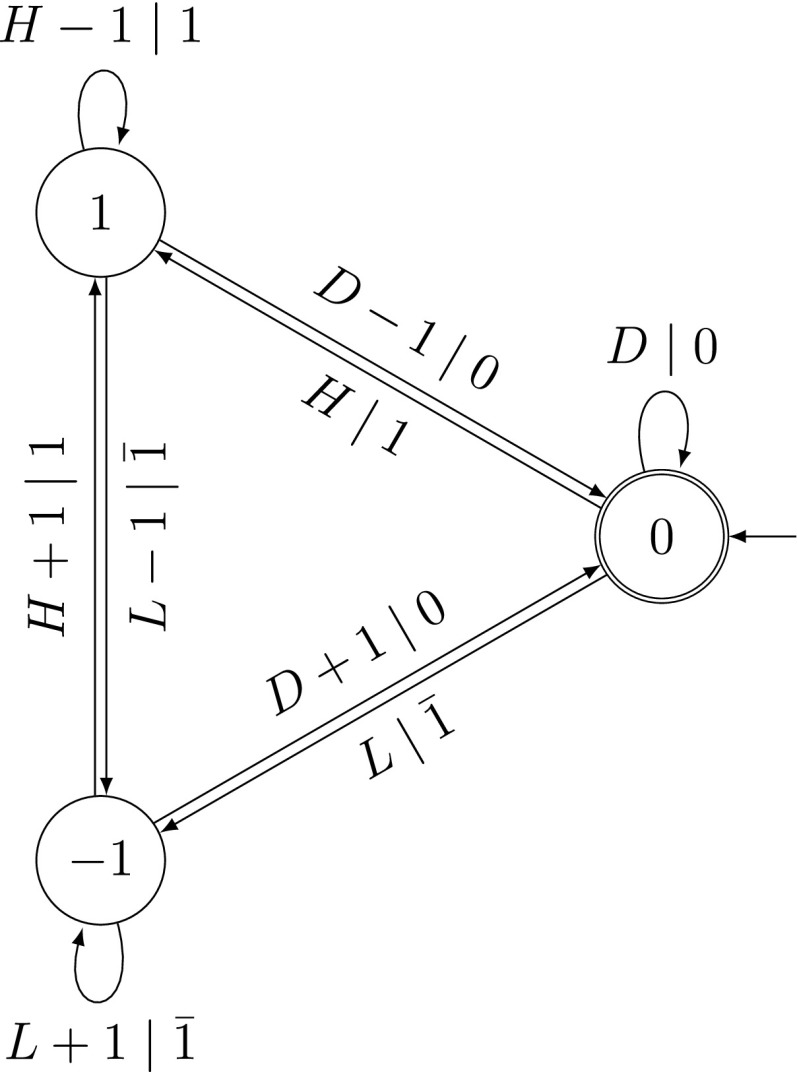
Standard addition for two (*q*, *d*)-expansions

The transducer in Fig. 1 computes the carries as in Algorithm 1. We use the sets $L=\{2d,\ldots ,d-1\}$, $D=\{d,\ldots ,q+d-1\}$ and $H=\{q+d,\ldots ,2q+2d-2\}$.

The transitions are constructed by using Algorithm 1 for the current input and carry.

#### Standard addition for SSDEs

**Fig. 2:**
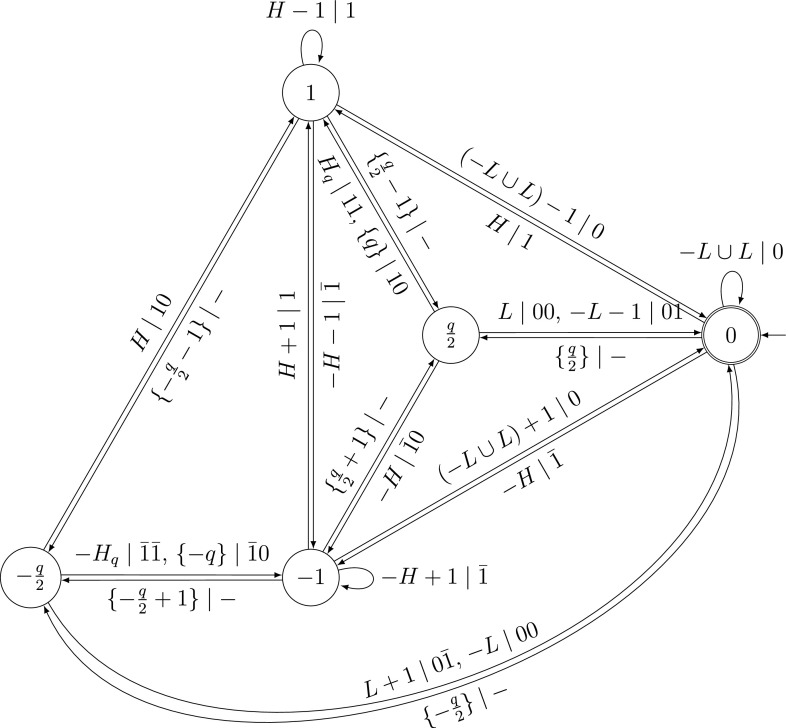
Standard addition for two SSDEs

The transducer in Fig. 2 computes the carries as in Algorithm 2. We use the sets $L=\{0,\ldots ,\frac{q}{2}-1\}$, $H=\{\frac{q}{2}+1,\ldots ,q\}$ and $H_{q}=\{\frac{q}{2},\ldots ,q-1\}$.

The transitions are constructed by using Algorithm 2 for the current input and carry.

The labels of the states $-1$, 0 and 1 encode the current carry. In the states with labels $\pm \frac{q}{2}$, we do not know yet whether the digit of the sum should be $\frac{q}{2}$ or $-\frac{q}{2}$ and thus, which carry is produced. To decide this, we have to look at the next digit. Thus, the transitions leading to a state $\pm \frac{q}{2}$ have no output (−) and the transitions starting at a state $\pm \frac{q}{2}$ have two output digits.

## Approximate equidistribution

As a probabilistic input model, we want to use an equidistribution on all digit expansions satisfying certain syntactical rules. This is easy in the case of (*q*, *d*)-expansions (see Sect. 4.1) because there are no syntactical rules. But in the case of a general regular language, like the SSDE, we can only approximate an equidistribution by Lemma 4.1. However, this approximation does not influence the main terms of the results.

A regular language is recognized by an automaton. An automaton is defined to consist of states, transitions between these states with labels, an initial state and final states. So to say, it is a transducer without output. The automaton recognizes a word from a language, if there exists a path starting at the initial state, leading to a final state with this word as the label.

We call an automaton aperiodic if its underlying directed graph is aperiodic, i.e., the greatest common divisor of all lengths of directed cycles of the graph is 1. If the underlying directed graph is strongly connected, then the automaton is so, too. If an automaton is strongly connected and aperiodic, then the adjacency matrix of the underlying graph is primitive.

Given an automaton $\mathcal {A}$ for a regular language, we automatically construct transition probabilities between the states to obtain an approximate equidistribution on all words of given length $\ell$. The weight of the word is the product of the transition probabilities multiplied with an *exit weight* [the factor in front of the product in (2) below]. This corresponds to an approximate equidistribution on all paths of length $\ell$ of the underlying graph of the automaton starting in the initial state. Without the exit weights, these transition probabilities are the same as defined by Shannon in [14] and Parry in [10]. We implemented the computations of this lemma as part of SageMath [13] as Automaton.shannon_parry_markov_chain.

### Lemma 4.1

Let $\mathcal {A}$ be a deterministic automaton with set of states $\{1,\ldots , n\}$, initial state 1, final states $\emptyset \ne F\subseteq \{1,\ldots , n\}$ recognizing a regular language $\mathcal {L}$. We assume that the adjacency matrix *A* of the underlying graph of $\mathcal {A}$ is primitive.

The dominant eigenvalue of *A* is denoted by $\lambda$, all other eigenvalues of *A* are assumed to be of modulus less than or equal to $\xi \lambda$ for some $0<\xi <1$. If there are eigenvalues of modulus $\xi \lambda$, then each of them must be semisimple, i.e., its algebraic and geometric multiplicities coincide.

Let $w>0$ and $u>0$ be right and left eigenvectors of *A* for the eigenvalue $\lambda$, respectively, such that $w_1=1$ and $\langle u,w\rangle =1$.

For a transition *t* from some state *i* to some state *j*, we set

1 $$\begin{aligned} p_{t}=\frac{w_j}{w_i \lambda }. \end{aligned}$$

For $\ell \ge 0$, the set of words of $\mathcal {L}$ of length $\ell$ is denoted by $\mathcal {L}_\ell$. For a word $x\in \mathcal {L}_\ell$, we denote the states and transitions used when $\mathcal {A}$ reads *x* by $1=s_0$, ..., $s_\ell$ and $t_1$, ..., $t_\ell$, respectively. The weight $W_{\ell }(x)$ of *x* is then defined to be

2 $$\begin{aligned} W_{\ell }(x)=\frac{1}{w_{s_\ell } \langle u, e_F\rangle }\prod _{j=1}^{\ell } p_{t_j} \end{aligned}$$

where $e_F$ is the indicator vector of the set *F* of final states.

Then

3 $$\begin{aligned} \sum _{t\text { leaves }i}p_{t}=1 \end{aligned}$$

holds for all states *i* and

4 $$\begin{aligned} W_{\ell }(x)=\frac{1}{|\mathcal {L}_\ell |}(1+O(\xi ^\ell )) \end{aligned}$$

holds uniformly for $\ell \ge 0$ and $x\in \mathcal {L}_\ell$.

Furthermore, consider the time-homogeneous Markov chain $\mathcal {M}$ on the state space $\{1,\ldots ,n\}$ where the transition probability from state *i* to state *j* is $\sum _{t}p_t$ where the sum runs over all transitions in $\mathcal {A}$ from *i* to *j*. Then this Markov chain has the stationary distribution

5 $$\begin{aligned} (u_1w_1, \ldots , u_nw_n). \end{aligned}$$

For large $\ell$ and a transition *t* from some state *i* to some state *j*, $p_t$ can be thought as the probability of using *t* under the condition that the automaton is currently in state *i*. Note that the sum in (3) runs over all transitions leaving *i* such that multiple transitions between *i* and *j* are counted separately although their individual weights $p_t$ only depend on *i* and *j*. It turns out that the exit weights do not influence the main term of our asymptotic expressions.

### *Proof of Lemma *4.1

We first note that the cardinality $|\mathcal {L}_\ell |$ is given by

$$\begin{aligned} |\mathcal {L}_\ell |=e_1^\top A^\ell e_F = \langle e_1,w\rangle \langle u,e_F\rangle \lambda ^{\ell }(1+O(\xi ^\ell ))=\langle u,e_F\rangle \lambda ^{\ell }(1+O(\xi ^\ell )) \end{aligned}$$

where $$e_1=(1,0,\ldots , 0)$$.

For $x\in \mathcal {L}_\ell$ with associated sequence of states $(s_0,\ldots , s_\ell )$, we have

$$\begin{aligned} W_{\ell }(x)=\frac{1}{w_{s_\ell } \langle u, e_F\rangle }\prod _{j=1}^{\ell } \frac{w_{s_j}}{w_{s_{j-1}} \lambda } =\frac{1}{w_{s_0} \langle u, e_F\rangle \lambda ^\ell }. \end{aligned}$$

As $w_{s_0}=w_1=1$, we get (4).

Next, we prove (3) by rewriting the sum as

$$\begin{aligned} \sum _{t\text { leaves }i}p_{t}=\sum _{j=1}^{n} a_{ij}\frac{w_j}{w_i \lambda }=1 \end{aligned}$$

by definition of *w*.

Finally, the transition matrix of the Markov chain $\mathcal {M}$ is

$$\begin{aligned} P=\Bigl (a_{ij}\frac{w_j}{w_i\lambda }\Bigr )_{1\le i,j\le n} \end{aligned}$$

by definition of the Markov chain and (1). Thus

$$\begin{aligned} P=\frac{1}{\lambda }{{\mathrm{diag}}}\Bigl (\frac{1}{w_1}, \ldots , \frac{1}{w_n}\Bigr ) A {{\mathrm{diag}}}(w_1, \ldots , w_n). \end{aligned}$$

As

$$\begin{aligned} (u_1w_1, \ldots , u_n w_n)P&= \frac{1}{\lambda }(u_1, \ldots , u_n)A{{\mathrm{diag}}}(w_1, \ldots , w_n)\\&= (u_1, \ldots , u_n){{\mathrm{diag}}}(w_1, \ldots , w_n)\\&=(u_1w_1, \ldots , u_n w_n), \end{aligned}$$

$(u_1w_1, \ldots u_n w_n)$ is a left eigenvector of *P* to the eigenvalue 1. By definition of *u* and *w*, $\sum _{i=1}^n u_iw_i=1$. As $\mathcal {M}$ is aperiodic and irreducible, $(u_1w_1, \ldots , u_nw_n)$ is the unique left eigenvector with this property and therefore the stationary distribution. $\square$

The weight $W_{\ell }$ induces a measure on the words of length $\ell$. The total measure of all words of length $\ell$ is 1 up to an exponentially small error, thus it is a probability measure up to an exponentially small error. Each word has exactly the same weight. If we see the transition probabilities as a part of the automaton, we obtain a probabilistic automaton:

### Definition 4.2

A probabilistic automaton is an automaton together with a map $p:t\mapsto p_t$ from the set of transitions to the interval [0, 1] such that

$$\begin{aligned} \sum _{t\text { leaves }s}p_{t}=1 \end{aligned}$$

holds for all states *s*. We call $p_t$ the weight or the probability of the transition *t*.

### Weights for (*q*, *d*)-expansions

We can use Lemma 4.1 in this case, too, but the digits of a (*q*, *d*)-expansion are independent of each other because there are no syntactical rules involving more than one digit. Therefore we can directly obtain equidistribution, not only approximating it. We first describe the direct way and later, in Remark 4.3, we consider using Lemma 4.1.

**Fig. 3:**
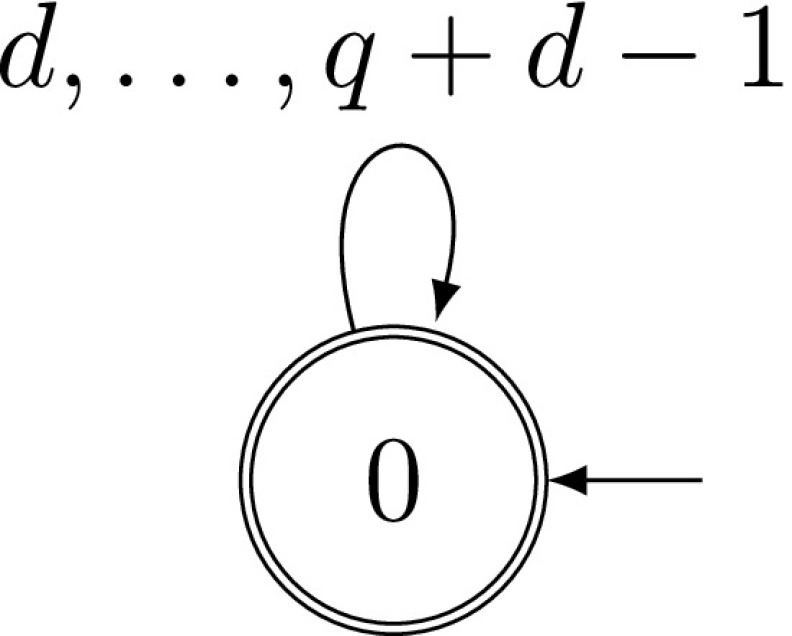
Automaton recognizing (*q*, *d*)-expansions

For any digit $x_{0}\in D$, we use the weights $W_{\ell }(x_{0})=\frac{1}{q}$. The exit weight is 1. By independence, we have the weight

$$\begin{aligned} W_{\ell }(x)=\frac{1}{q^{\ell }} \end{aligned}$$

for a digit expansion *x* of length $\ell$. With this weight, we have an equidistribution of all (*q*, *d*)-expansions of length $\ell$.

#### Remark 4.3

The same weights can be obtained by Lemma 4.1. The transition probabilities are $p_{0\rightarrow 0}=q^{-1}$. As the automaton recognizing (*q*, *d*)-expansions has only one state (see Fig. 3), there is no error term in (4).

**Fig. 4:**
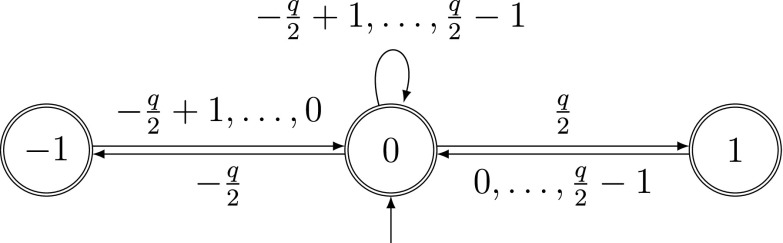
Automaton recognizing SSDEs

### Weights for SSDEs

The automaton in Fig. 4 recognizes SSDEs. The adjacency matrix of this automaton is

$$\begin{aligned} A=\begin{pmatrix}0&{}\quad \frac{q}{2}&{}\quad 0\\ 1&{}\quad q-1&{}\quad 1\\ 0&{}\quad \frac{q}{2}&{}\quad 0\end{pmatrix} \end{aligned}$$

where the states are ordered by their labels.

The matrix *A* has the eigenvalues *q*, $-1$ and 0. The vectors $(\frac{1}{q+1},\frac{q}{q+1},\frac{1}{q+1})$ and $(\frac{1}{2},1,\frac{1}{2})^{\top }$ are the left and right eigenvector corresponding to the eigenvalue *q*, respectively. The transition probabilities are

6 $$\begin{aligned} p_{-1\rightarrow 0}&=p_{1\rightarrow 0}=\frac{2}{q},&\quad p_{0\rightarrow 1}&=p_{0\rightarrow -1}=\frac{1}{2q},&\quad p_{0\rightarrow 0}&=\frac{1}{q}. \end{aligned}$$

The constant in the error term is $\xi =\frac{1}{q}$. The exit weights are $(2,1,2)\cdot \frac{q+1}{q+2}$.

With these transition probabilities, the asymptotic frequencies of the digits (cf. [6]) can be computed as

7 $$\begin{aligned} {\left\{ \begin{array}{ll} \frac{1}{2(q+1)}&{}\text {if the digit is }\pm \frac{q}{2},\\ \frac{q+2}{q(q+1)}&{}\text {if the digit is }0,\\ \frac{1}{q}&{}\text {otherwise} \end{array}\right. } \end{aligned}$$

by using the stationary distribution given in (5).

## Asymptotic analysis of the standard addition

In this section, we use the probabilistic model defined in Sect. 4 for the input sequence of the transducers in Sect. 3.2. Then we will use Lemma 5.1 to obtain expectation, variance and asymptotic normality of the number of carries.

In Sects. 5.1 and 5.2, we will construct probabilistic automata whose transition labels are the carries and where each transition has a weight corresponding to the weight constructed in Sect. 4.

Let *m* and *n* be two functions mapping the output of a transition into the real numbers; for brevity we write *m*(*t*) and *n*(*t*) without mentioning the output label of the transition *t*. In our setting *m* will count the number of carries 1, and *n* the number of carries $-1$ of the output of a transition. We consider the two random variables $M_{\ell }$ and $N_{\ell }$ which are the sum of the values of *m* and *n*, respectively, over a path of length $\ell$ with probability the product of the weights of this path multiplied with the exit weight.

We will use multivariate generating functions in three variables *x*, *y* and *z*. The variables *x* and *y* mark the number of carries 1 and $-1$, respectively, and the variable *z* marks the length of the expansion.

The transition matrix *A*(*x*, *y*) of a probabilistic automaton with *K* states and two functions *m* and *n* is a $K\times K$ matrix whose (*i*, *j*)-th entry is

$$\begin{aligned} \sum _{t:i\rightarrow j}p_{t}x^{m(t)}y^{n(t)} \end{aligned}$$

where $p_{t}$ is the weight of the transition *t*.

The next lemma is a slight modification of [5, Theorem 3.9] taking into account the non-uniform distribution of the input alphabet.

### Lemma 5.1

Let $\mathcal A$ be a strongly connected, aperiodic probabilistic automaton where all states are final. Let *m* and *n* be functions mapping the output of a transition into the real numbers and *A*(*x*, *y*) be the associated transition matrix of the automaton, where *x* and *y* mark *m* and *n*, respectively. Let $M_\ell$ and $N_\ell$ be the associated random variables as defined above.

Define the function $f(x,y,z)=\det (I-zA(x,y))$. Then the expected value of $(M_{\ell },N_{\ell })$ is $(e_{m}, e_{n})\ell +\mathcal {O}(1)$ with

$$\begin{aligned} e_{m}&=\left. \frac{f_{x}}{f_{z}}\right| _{(1,1,1)},\\ e_{n}&=\left. \frac{f_{y}}{f_{z}}\right| _{(1,1,1)}. \end{aligned}$$

The variance-covariance matrix $$\big ({\begin{matrix}v_{m}&{}c\\ c&{}v_{n}\end{matrix}}\big )\ell +\mathcal {O}(1)$$ has the entries

8 $$\begin{aligned} v_{m}=\left. \frac{f_{x}^{2}(f_{zz}+f_{z})+f_{z}^{2}(f_{xx}+f_{x})-2f_{x}f_{z}f_{xz}}{f_{z}^{3}}\right| _{(1,1,1)},\end{aligned}$$

9 $$\begin{aligned} v_{n}=\left. \frac{f_{y}^{2}(f_{zz}+f_{z})+f_{z}^{2}(f_{yy}+f_{y})-2f_{y}f_{z}f_{yz}}{f_{z}^{3}}\right| _{(1,1,1)},\end{aligned}$$

10 $$\begin{aligned} c=\left. \frac{f_{x}f_{y}(f_{zz}+f_{z})+f_{z}^{2}f_{xy}-f_{y}f_{z}f_{xz}-f_{x}f_{z}f_{yz}}{f_{z}^{3}}\right| _{{(1,1,1)}}. \end{aligned}$$

Furthermore, if $v_{m}$ and $v_{n}$ are non-zero, then $M_{\ell }$ and $N_{\ell }$ are asymptotically normally distributed, respectively. If the variance-covariance matrix is non-singular, then $M_{\ell }$ and $N_{\ell }$ are asymptotically jointly normally distributed.

### Proof

The moment generating function is

$$\begin{aligned} \mathbb E\exp (s_{1}M_{\ell }+s_{2}N_{\ell })=[z^{\ell }]e_{1}^{\top }(I-zA(e^{s_{1}},e^{s_{2}}))^{-1}w_{F} \end{aligned}$$

where $e_{1}$ is a unit vector with a 1 at the position of the initial state and the entries of $w_{F}$ are the exit weights of the states. Since the automaton is probabilistic and aperiodic, the unique dominant eigenvalue of *A*(1, 1) is 1. Thus the same arguments apply as in [5] after replacing “complete” by “probabilistic”. We obtain the same formulas for the constants of the expectation, the variance and the covariance. Also the central limit theorem follows. $\square$

### Standard addition for (*q*, *d*)-expansions

To construct the probabilistic automaton, we start with the transducer in Fig. 1, and use the weights from Sect. 4.1.

All steps in this section, including the computation of the constants in Theorem 1, can be done in the mathematical software system SageMath [13] by using the included finite state machine package described in [3]. The corresponding SageMath file is available at [4].

The construction in this section is more general than needed for the case of independent digits as in (*q*, *d*)-expansions. But discussing it here in full generality allows reusing the same ideas for the case of dependent digits as in SSDEs later on. We will use the same construction for SSDEs in Sects. 5.2 and 7.

In this section, let $\mathcal {A}$ be the automaton in Fig. 3, equipped with the weight $\frac{1}{q}$ for every transition and the exit weight 1 for every state (by Sect. 4.1). Construct $\mathcal {A}^{2}$ as the *additive Cartesian product* 1 of $\mathcal {A}$ with itself by the following rules:

- The states of $\mathcal {A}^{2}$ are pairs of states of $\mathcal {A}$.
- There is a transition from (*a*, *b*) to (*c*, *d*) with label $x+y$ in $\mathcal {A}^{2}$ if there are transitions from *a* to *c* with label *x* and *b* to *d* with label *y* in $\mathcal {A}$.
- The weight of a transition in $\mathcal {A}^{2}$ is the product of the weights of the two transitions in $\mathcal {A}$.
- The exit weight of a state in $\mathcal {A}^{2}$ is the product of the exit weights of the two states in $\mathcal {A}$.

The probabilistic automaton $\mathcal {A}^{2}$ recognizes all possible sequences $\varvec{s}$ of digitwise sums with the correct weights for the equidistribution on the independent (*q*, *d*)-expansions $\varvec{x}$ and $\varvec{y}$.

In this section, let $\mathcal {B}$ be the transducer in Fig. 1 performing the standard addition of two (*q*, *d*)-expansions. Next, we construct $\mathcal {S}_{(q,d)}$ as the composition $\mathcal {B}\circ \mathcal {A}^{2}$ by the following rules:

- The states of $\mathcal {S}_{(q,d)}$ are pairs of states of $\mathcal {B}$ and $\mathcal {A}^{2}$.
- For each pair of transitions from *a* to *c* with input label *s* and output label *k* in $\mathcal {B}$ and from *b* to *d* with weight *w* and label *s* in $\mathcal {A}^{2}$, there is a transition from (*a*, *b*) to (*c*, *d*) with weight *w* and label *k* in $\mathcal {S}_{(q,d)}$.
- The exit weight of a state in $\mathcal {S}_{(q,d)}$ is the exit weight of the corresponding state in $\mathcal {A}^{2}$.

The probabilistic automaton $\mathcal {S}_{(q,d)}$ recognizes the sequence of carries $\varvec{c}$ with the correct weights for the equidistribution on the independent (*q*, *d*)-expansions $\varvec{x}$ and $\varvec{y}$. The probabilistic automaton $\mathcal {S}_{(q,d)}$ has three states.

To determine the transition matrix of $\mathcal {S}_{(q,d)}$, we use the following lemma to compute the number of transitions between two states. The lemma is proved by an inclusion-exclusion argument.

#### Lemma 5.2

Let

$$\begin{aligned}&N(x_{\min },x_{\max },y_{\min },y_{\max },s_{\min },s_{\max })\\&\quad =|\{(x,y)\in \mathbb Z^{2}\mid x_{\min }\le x\le x_{\max }, y_{\min }\le y\le y_{\max }, s_{\min }\le x+y\le s_{\max }\}|. \end{aligned}$$

Then we have

$$\begin{aligned} {}&N(x_{\min },x_{\max },y_{\min },y_{\max },s_{\min },s_{\max })\\&\quad =N(0,\infty ,0,\infty ,0,s_{\max }-x_{\min }-y_{\min })\\&\quad \quad \,-\,N(0,\infty ,0,\infty ,0,s_{\max }-x_{\min }-y_{\max }-1)\\&\quad \quad \,-\,N(0,\infty ,0,\infty ,0,s_{\max }-x_{\max }-y_{\min }-1)\\&\quad \quad \,+\,N(0,\infty ,0,\infty ,0,s_{\max }-x_{\max }-y_{\max }-2)\\&\quad \quad \,-\,N(0,\infty ,0,\infty ,0,s_{\min }-x_{\min }-y_{\min }-1)\\&\quad \quad \,+\,N(0,\infty ,0,\infty ,0,s_{\min }-x_{\min }-y_{\max }-2)\\&\quad \quad \,+\,N(0,\infty ,0,\infty ,0,s_{\min }-x_{\max }-y_{\min }-2)\\&\quad \quad \,-\,N(0,\infty ,0,\infty ,0,s_{\min }-x_{\max }-y_{\max }-3) {}\end{aligned}$$

with $N(0,\infty ,0,\infty ,0,s_{\max })=0$ if $s_{\max }$ is negative and

$$\begin{aligned} N(0,\infty ,0,\infty ,0,s_{\max })=\frac{1}{2}(s_{\max }+2)(s_{\max }+1) {}\end{aligned}$$

otherwise.

This gives the transition matrix in Table 6 in the appendix where *x* marks carries 1 and *y* marks carries $-1$. For example, the entry in the first row and column is

$$\begin{aligned} \frac{(d-1)(d-2)}{2q^{2}}y=\sum _{\begin{array}{c} x,y\in D\\ x+y\in L+1 \end{array}}p_{0\rightarrow 0}p_{0\rightarrow 0}y=\frac{1}{q^{2}}N(d,q+d-1,d,q+d-1,2d,d)y \end{aligned}$$

because this entry corresponds to the transitions from $-1$ to $-1$ with input label $L+1$ and output label $\bar{1}$ in $\mathcal {B}$ and from (0, 0) to (0, 0) in $\mathcal {A}^{2}$.

With the transition matrix, the next theorem follows directly from Lemma 5.1.

#### Theorem 1

Let $M_{\ell }$ and $N_{\ell }$ be the number of carries 1 and $-1$, respectively, when adding two independent random (*q*, *d*)-expansions of length $\ell$. The expected value of $(M_{\ell },N_{\ell })$ is $(e_{1},e_{-1})\ell +\mathcal {O}(1)$ with constants

$$\begin{aligned} e_{1}&=\frac{(q + d - 1)^2}{2(q - 1)^2},\\ e_{-1}&=\frac{d^{2}}{2(q-1)^{2}}. \end{aligned}$$

The variance-covariance matrix of $(M_{\ell },N_{\ell })$ is $$\big ({\begin{matrix}v_{1}&{}c\\ c&{}v_{-1}\end{matrix}}\big )\ell +\mathcal {O}(1)$$ with constants

$$\begin{aligned} v_{1}&=\frac{(q + d - 1)^2 (q^4 - 2q^3d - q^2d^2 - 4qd^2 - 2q^2 - d^2 + 2d + 1)}{4(q - 1)^5 (q + 1)},\\ v_{-1}&=\frac{d^{2}(2q^4 - q^2d^2 - 4q^3 - 6q^2d - 4qd^2 + 4q^2 + 6qd - d^2 - 4q + 2)}{4(q - 1)^5 (q + 1)},\\ c&=\frac{d(q+d-1)(q^3d + q^2d^2 - q^3 + 3q^2d + 4qd^2 + 2q^2 - 3qd + d^2 - q - d)}{4(q - 1)^5 (q + 1)}. \end{aligned}$$

Furthermore, the number of carries 1 and $-1$ is asymptotically jointly normally distributed for $d\ne 0$, $-q+1$. For $d=0$, $M_\ell$ is asymptotically normally distributed and $N_\ell =0$ because the carry $-1$ does not occur. For $d=-q+1$, the same holds with $M_\ell$ and $N_\ell$ exchanged.

**Fig. 5:**
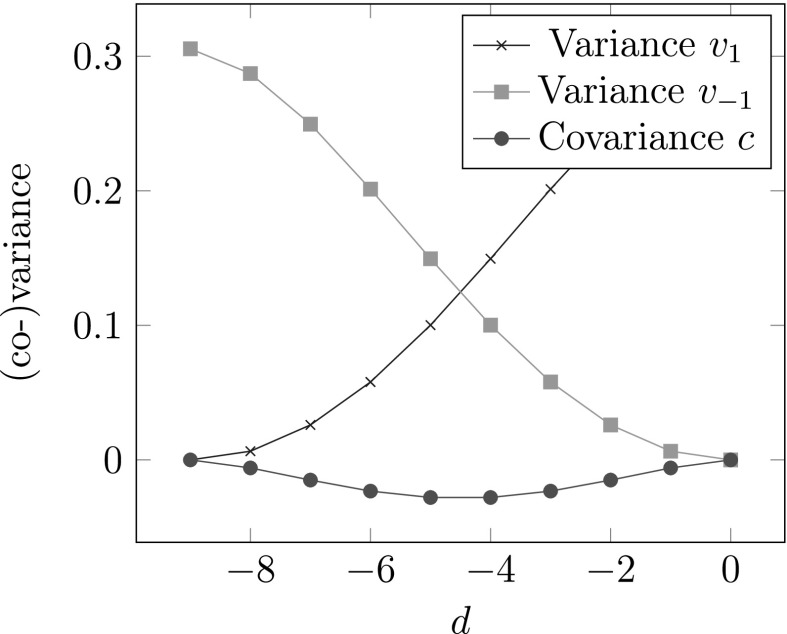
Variances and covariance for (10, *d*)-expansions of Theorem 1

In Fig. 5, variances and covariance for (10, *d*)-expansions are shown.

#### Remark 5.3

The expected value for carries in the addition of (*q*, *d*)-expansions corresponds to the result in [9]. There, the authors find the stationary distribution

$$\begin{aligned} \frac{1}{2(q-1)^{2}}(d^{2},\, q^{2}-2q+1-2qd+2d-2d^{2},\, (q+d-1)^{2}) \end{aligned}$$

for the states $(-1,0,1)$ of the carry process. For $d=\frac{-q+1}{2}$, this stationary distribution can also be found in [1].

### Standard addition for SSDEs

To cope with the dependencies between the digits, we have to combine the conditional probabilities of the automaton in Fig. 4 with the carries computed by the automaton in Fig. 2. This is done in the same way as in Sect. 5.1.

All steps in this section, including the computation of the constants in Theorem 2, can be done in the mathematical software system SageMath [13] by using its included finite state machine package described in [3]. The corresponding SageMath file is available at [4].

In this section, let $\mathcal {A}$ be the automaton in Fig. 4 equipped with the weights in (6) and let $\mathcal {B}$ be the transducer in Fig. 2 performing the standard addition of two SSDEs.

We first construct the additive Cartesian product $\mathcal {A}^{2}$, recognizing all possible sequences $\varvec{s}$ of digitwise sums with the correct weights approximating the equidistribution on two independent SSDEs $\varvec{x}$ and $\varvec{y}$. This probabilistic automaton has 9 states.

Next, we construct $\mathcal {S}_{\text {SSDE}}$ as the composition $\mathcal {B}\circ \mathcal {A}^{2}$. This probabilistic automaton recognizes the sequence of carries $\varvec{c}$ with the correct weights approximating the equidistribution on two independent SSDEs $\varvec{x}$ and $\varvec{y}$. This gives a transducer with 45 states.

Because of symmetries (cf. Remark 3.1), we can simplify $\mathcal {S}_{\text {SSDE}}$ such that it has only 14 states^2^:

#### Lemma 5.4

A probabilistic automaton can be simplified by applying the following rules:

- If between two states, there are two transitions with the same label, then these two transitions can be combined. The weights are summed up in this process.
- Let $\{C_{1},\ldots ,C_{k}\}$ be a partition of the states of the automaton with the following property: If *a*, $b\in C_{j}$ are two states, then there is a bijection between the transitions leaving *a* and the ones leaving *b* which preserves the label, the weight of the transition and into which set of the partition the transitions lead. These bijections define an equivalence relation on the transitions leaving a set of the partition. Then each set of the partition can be contracted to a new state. For each equivalence class of transitions, there is one transition in the simplified transducer.

Thus, we obtain a $14\times 14$ transition matrix of $\mathcal {S}_{\text {SSDE}}$ given in Table 7 in the appendix (using Lemma 5.2).

#### Theorem 2

The expected value of the number of carries equal to 1 when adding two SSDEs of length $\ell$ is

$$\begin{aligned} \frac{q^{2} + 2 q + 4}{8(q + 1)^{2}} \ell +\mathcal {O}(1) \end{aligned}$$

and the variance is

$$\begin{aligned} \frac{7 q^{6} + 48 q^{5} + 159 q^{4} + 128 q^{3} - 48 q^{2} - 12 q - 8}{64(q + 1)^{5} (q - 1)}\ell +\mathcal {O}(1). \end{aligned}$$

The same result holds for carries equal to $-1$. The covariance between carries 1 and $-1$ is

$$\begin{aligned} -\frac{q^6 + 24q^5 + 33q^4 + 80q^3 + 120q^2 - 12q - 8}{64(q + 1)^{5} (q - 1)}\ell +\mathcal {O}(1). \end{aligned}$$

The number of carries 1 and $-1$ is asymptotically jointly normally distributed.

**Fig. 6:**
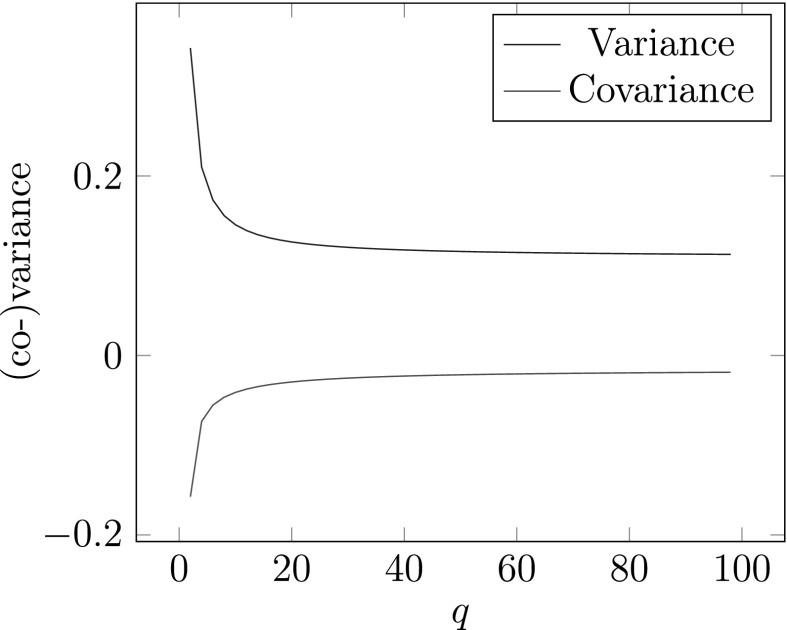
Variance and covariance for SSDEs for $q=2,\ldots ,100$ of Theorem 2

In Fig. 6, variance and covariance for SSDEs are shown.

#### Proof

We can compute the determinant $f(x,y,z)=\det (I-zA(x,y))$ of the transition matrix *A*(*x*, *y*) in the appendix of the simplified automaton $\mathcal {S}_{\text {SSDE}}$ with 14 states. Thus, Lemma 5.1 implies the expected value, the variance and the central limit theorem where the input sequence is the sum of two independent SSDEs of length $\ell$ with the approximate equidistribution $W_{\ell }$.

As the (exact) equidistribution $\mathbb P_{\ell }$ satisfies $\mathbb P_{\ell }=(1+\mathcal {O}(\xi ^{\ell }))W_{\ell }$, these results also hold for the (exact) equidistribution. $\square$

#### Remark 5.5

If we neglect the dependencies between two adjacent digits, we obtain a different result: Assume that the digits are independently distributed with probabilities given in (7). Then the expected value of the number of carries 1 is

$$\begin{aligned} \frac{q^{12} + 7 q^{11} + 19 q^{10} + 27 q^{9} + 24 q^{8} + 9 q^{7} - 15 q^{6} - 15 q^{5} + 47 q^{4} + 104 q^{3} + 64 q^{2} - 48 q - 48}{8(q + 1)^{3} q^{2} (q^{7} + 4 q^{6} + 5 q^{5} - q^{4} - 9 q^{3} - 8 q^{2} + 4)} \end{aligned}$$

and the variance is

$$\begin{aligned}&\frac{1}{64}(7 q^{38} + 152 q^{37} + 1557 q^{36} + 9958 q^{35} + 44300 q^{34} + 144166 q^{33} + 349511 q^{32}\\&\qquad + 622942 q^{31} + 756995 q^{30} + 432788 q^{29} - 439628 q^{28} - 1347486 q^{27} - 1407649 q^{26} \\&\qquad - 466340 q^{25}- 39181 q^{24} - 2293904 q^{23} - 6902413 q^{22} - 9055044 q^{21} - 2972395 q^{20} \\&\qquad + 10157788 q^{19} + 19040707 q^{18}+ 12034998 q^{17} - 7655356 q^{16} - 21471482 q^{15} \\&\qquad - 15688011 q^{14}+ 1495584 q^{13} + 10611092 q^{12} + 5762536 q^{11}- 1482784 q^{10} \\&\qquad - 1794016 q^{9} + 1000784 q^{8} + 744768 q^{7}- 1199872 q^{6} - 1204224 q^{5} + 120832 q^{4} \\&\qquad + 574464 q^{3}+ 172032 q^{2} - 73728 q - 36864)\\&\times q^{-4}(q + 1)^{-6} (q^{7} + 4 q^{6} + 5 q^{5} - q^{4} - 9 q^{3} - 8 q^{2} + 4)^{-3}\\&\times (q^{7} + 2 q^{6} + q^{5} + q^{4} + q^{3} - 2 q^{2} + 4)^{-1}. \end{aligned}$$

As expected, the limit for *q* to infinity is the same.

## Von Neumann’s addition

In this section, we analyze von Neumann’s addition algorithm for SSDEs, a parallel algorithm using several iterations. This algorithm was analyzed by Knuth in [8] for standard *q*-ary expansions. In [7], this analysis was extended to (*q*, *d*)-expansions and SSDEs. However, for $q\ge 4$, the hardware and software available at that time made the use of the probabilistic model of Sect. 4.2 computationally infeasible. The approximate model described in Remark 5.5 was used instead. As Remark 5.5 demonstrates, this approximation may lead to different main terms in the expectation and the variance.

**Fig. 7:**
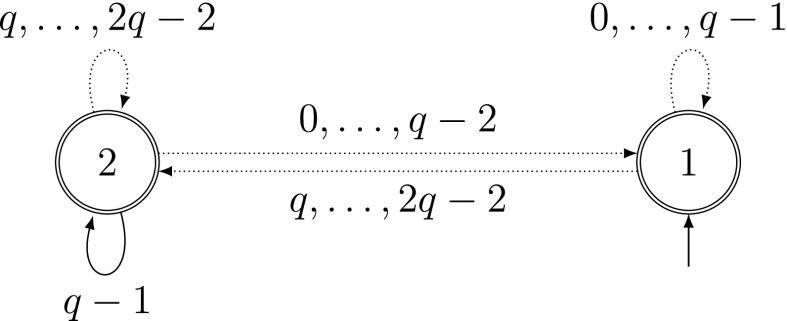
Automaton to find the longest carry generating sequence for von Neumann’s addition of two standard *q*-ary expansions

As before, we choose an approximate equidistribution for all independent pairs of SSDEs of length $\ell$ as our probabilistic input model. In contrast to the result in [7], we obtain more natural constants occurring in the main term of the expectation and the variance.

For von Neumann’s addition of two standard *q*-ary digit expansions, the number of iterations depends on the longest subsequence $(q-1)\ldots (q-1)j$ with $j\ge q$ of the digitwise sum $\varvec{s}$, see [8]. Such sequences can be found by an automaton with two classes of transitions (see Fig. 7 and [7, Figure 1]). One class corresponds to the digit $(q-1)$ of a carry generating sequence and is depicted by solid lines. The other class corresponds to all other digits (including the digit *j* of a carry generating sequence) and is depicted by dotted lines. The longest consecutive run of solid edges in the automaton in Fig. 7 corresponds to the number of iterations of von Neumann’s addition minus 2. The asymptotic analysis of these longest runs can be performed using the probabilistic version of the automaton in Fig. 7. We will extend this approach to SSDEs with arbitrary even base using a larger probabilistic automaton in Sect. 7.

### Algorithm

**Table 5:**
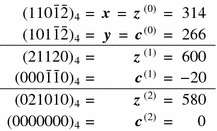
Example for von Neumann’s addition for SSDEs with $q=4$

We have $$t(110\bar{1}\bar{2},101\bar{1}\bar{2})=2$$

Let $\varvec{x}$ and $\varvec{y}$ be two SSDEs. The idea of the algorithm is to construct the sequence of digitwise sums $\varvec{s}=\varvec{x}+\varvec{y}$ and correct each position if the number at this position is not in the digit set or at the border of the digit set where we have to take into account the syntactical rule. In Table 5, an example for von Neumann’s addition for SSDEs with $q=4$ is given.

As in [7], we define $(\varvec{z},\varvec{c})={{\mathrm{add}}}(\varvec{s})$ with $\varvec{s}=\varvec{x}+\varvec{y}$ by

$$\begin{aligned} c_{0}&=0,\\ c_{j+1}&= {\left\{ \begin{array}{ll} {{\mathrm{sgn}}}(s_{j})&{}\text {if }|s_{j}|>\frac{q}{2},\\ &{}\quad \text {or }|s_{j}|=\frac{q}{2} \text { and } \\ &{}\quad ({{\mathrm{sgn}}}(s_{j})s_{j+1})\bmod q\ge \frac{q}{2}\\ 0&{}\text {otherwise,} \end{array}\right. }\\ z_{j}&=s_{j}-c_{j+1}q. \end{aligned}$$

Here, the choice of the carry $c_{j+1}$ corresponds to the one in Algorithm 2. By iterating this step we obtain $(\varvec{z}^{(k+1)},\varvec{c}^{(k+1)})={{\mathrm{add}}}(\varvec{z}^{(k)}+\varvec{c}^{(k)})$ with $\varvec{z}^{(0)}=\varvec{x}$ and $\varvec{c}^{(0)}=\varvec{y}$. If $\varvec{c}^{(k)}=0$, then $\varvec{z}^{(k)}$ is the SSDE of the sum $\varvec{x}+\varvec{y}$ and the algorithm stops. Note that during this process, $\varvec{z}^{(k)}$ and $\varvec{c}^{(k)}$ are not necessarily SSDEs.

In [7], the correctness and the termination of this algorithm were proved. We denote the number of iterations of von Neumann’s addition algorithm by $t(\varvec{x},\varvec{y})=\min \{k\ge 0: \varvec{c}^{(k)}=0\}$.

**Fig. 8:**
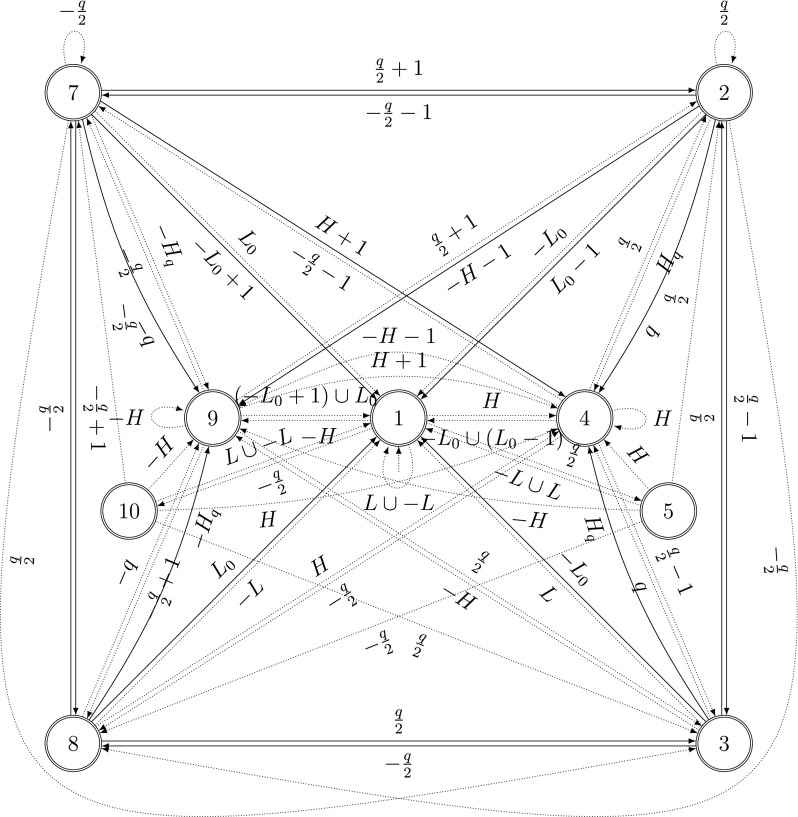
Automaton in [7, Figure 5]: $t(\varvec{x},\varvec{y})\le k+2$ if and only if the automaton traverses at most *k* solid edges when reading $(s_j)_{j\,\ge \, 0}$

### Automaton

A description of all SSDEs $\varvec{x}$ and $\varvec{y}$ with $t(\varvec{x},\varvec{y})=k$ is given in [7]. This description is in terms of an automaton and leads to the automaton in [7, Figure 5] reproduced here as Fig. 8. We use the sets $L=\{0,\ldots ,q/2-1\}$, $L_{0}=L\setminus \{0\}$, $H=\{q/2+1,\ldots , q\}$ and $H_{q}=H\setminus \{q\}$.

From [7, Theorem 3.4], we know that $t(\varvec{x},\varvec{y}) \le k+2$ if and only if this automaton traverses at most *k* consecutive solid transitions when reading $\varvec{s}$.

#### Remark 6.1

Strictly speaking, the automaton reads the sequence $(s_{j})_{j\ge 0}$ where $s_{j}=x_{j}+y_{j}$ for $j\le J$ and $s_{j}=0$ for $j>J$, for some *J*. However, most of the solid edges are visited while $j\le J$. All transitions with label 0 lead to state 1. Those from states 2 and 7 are solid edges, all others are dotted. If the transition is in state 2 (or 7) after reading $s_{J}$, an additional solid edge will be traversed. Thus, we have to specially treat the states 2 and 7.

## Asymptotic analysis of von Neumann’s addition

For the asymptotic analysis, we combine the automaton in Fig. 8 with the probabilistic model for SSDEs from Sect. 4.2 in the same way as in Sect. 5.2.

All steps in this section, including the computation of the constants in Theorem 4, can be done in the mathematical software system SageMath [13] by using the included finite state machine package described in [3]. The corresponding SageMath file is available at [4].

We again use the automata $\mathcal A$ and $\mathcal A^{2}$ described in Sect. 5.2, recognizing SSDEs and the digitwise sum of two SSDEs, respectively. As before, the next step is to construct the Cartesian product $\mathcal {N}_{\text {SSDE}}$ of the automaton $\mathcal {B}$ in Fig. 8 and $\mathcal A^{2}$.

After simplifying this construction as described in Lemma 5.4, the probabilistic automaton $\mathcal {N}_{\text {SSDE}}$ has 12 states:

11 $$\begin{aligned}&\{(1,(-1, 1)),(1,(1, -1))\},\nonumber \\&\{(4,(0, 0)), (9,(0, 0))\},\nonumber \\&\{(5,(0, 1)), (5,(1, 0)), (10,(-1,0)), (10,(0, -1))\},\nonumber \\&\{(2,(0, 1)), (2,(1, 0)), (7,(-1, 0)), (7,(0, -1))\},\nonumber \\&\{(5,(0, 0)),(10,(0, 0))\},\nonumber \\&\{(2,(0,0)), (7,(0, 0))\},\nonumber \\&\{(1,(-1, 0)), (1,(0, -1)), (1,(0, 1)), (1,(1, 0))\},\nonumber \\&\{(3,(0, 1)), (3,(1, 0)), (8,(-1, 0)),(8,(0, -1))\},\nonumber \\&\{(1,(0, 0))\},\nonumber \\&\{(3,(0, 0)), (8,(0, 0))\},\nonumber \\&\{(4,(1, 1)), (9,(-1, -1))\},\nonumber \\&\{(4,(0, 1)), (4,(1, 0)), (9,(-1, 0)),(9,(0, -1))\}. \end{aligned}$$

In this case, the simplification is done in the same way as in Lemma 5.4, but also taking into account the class (dotted or solid) of a transition. The partition of the set of states was constructed by the symmetries between the two sequences $\varvec{x}$ and $\varvec{y}$ described in Remark 3.1, for example $\{(1,(-1, 1)),(1,(1, -1))\}$, and the additional vertical symmetry of the automaton in Fig. 8, for example $\{(4,1, 1),(9,-1, -1)\}$.

The state (1, (0, 0)) is initial and all states are final.

The next theorem is an extension of Lemma 2.5 in [7] additionally including the variance and convergence in distribution.

### Theorem 3

Let $w_{\ell k}$, $\ell$, $k \ge 0$, be non-negative numbers with generating function

$$\begin{aligned} G_{k}(z)=\frac{R_k(z)}{S_k(z)}=\sum _{\ell \ge 0} w_{\ell k} z^\ell \end{aligned}$$

such that $w_{\ell k}$ is non-decreasing in *k*.

Assume that

$$\begin{aligned} R_k(z)&=r_0(z)+ r_{1}\Big (z,\Big (\frac{z}{a_{1}}\Big )^{k},\ldots ,\Big (\frac{z}{a_{m}}\Big )^{k}\Big ),\\ S_k(z)&=(1-z)s_0(z)+\Big (\frac{z}{a_1}\Big )^k s_1(z)+ s_{2}\Big (z,\Big (\frac{z}{a_{1}}\Big )^{k},\ldots ,\Big (\frac{z}{a_{m}}\Big )^{k}\Big ), \end{aligned}$$

where $r_0$, $s_0$, and $s_1$ are real polynomials in *z* (not depending on *k*). Furthermore, $r_{1}$ and $s_{2}$ are real polynomials in *z*, $(z/a_1)^k$, ..., $(z/a_m)^k$ for some $m\ge 2$ and some real numbers $1<a:=a_1<|a_2|\le |a_3|\le \dots \le |a_m|$ such that each of the summands in $r_{1}$ is divisible by one of the terms $(z/a_{1})^{k}$, ..., $(z/a_{m})^{k}$ and each of the summands in $s_{2}$ is divisible by one of the terms $(z/a_{1})^{2k}$, $(z/a_{2})^{k}$, ..., $(z/a_{m})^{k}$. Define

$$\begin{aligned} \delta :=s_1(1)/s_0(1),\qquad \rho :=\min \left( \log |a_2|/\log a_1, 2\right) -1. \end{aligned}$$

Assume furthermore that $r_{0}(1)\ne 0$, that $s_0$ does not have any zero in $|z|\le 1$ and that $\delta >0$.

Then $$G(z):=\frac{r_{0}(z)}{(1-z)s_{0}(z)}=\lim _{k\rightarrow \infty } G_k(z)$$ and

$$\begin{aligned} G(z)=\sum _{\ell \ge 0}w_{\ell }z^{\ell } \end{aligned}$$

with $w_{\ell }=w_{\ell k}$ for $k\ge \ell$. Additionally, $w_{\ell }\ne 0$ for $\ell \ge \ell _{0}$ for a suitable $\ell _{0}$.

Let $(X_{\ell })_{\ell \ge \ell _{0}}$ be the sequence of random variables with support $\mathbb N_{0}$ defined by

$$\begin{aligned} \mathbb P(X_{\ell }\le k)=\frac{w_{\ell k}}{w_{\ell }}. \end{aligned}$$

Then the asymptotic formula

12 $$\begin{aligned} \frac{w_{\ell k}}{w_{\ell }}=\exp (-\delta \ell /a^{k})(1+o(1)) \end{aligned}$$

holds as $\ell \rightarrow \infty$ for $k=\log _{a} \ell +\mathcal {O}(1)$. Hence the shifted random variable $X_{\ell }-\log _{a}\ell$ converges weakly to a limiting distribution if $\ell$ runs through a subset of the positive integers such that the fractional part $\{\log _{a}\ell \}$ of $\log _{a}\ell$ converges.

The expected value of $X_{\ell }$ is

13 $$\begin{aligned} \mathbb EX_{\ell }=\log _a\ell + \log _a\delta +\frac{\gamma }{\log a}+\frac{1}{2}+ \Psi _{0}(\log _a \ell +\log _a\delta ) + \mathcal {O}\left( \frac{\log ^{\rho +3} \ell }{\ell ^\rho }\right) , \end{aligned}$$

the variance is

14 $$\begin{aligned}&\mathbb VX_{\ell }=\frac{\pi ^{2}}{6\log ^{2}a}+\frac{1}{12}+\Psi _{1}(\log _{a}\ell +\log _{a}\delta )-\frac{2\gamma }{\log a}\Psi _{0}\left( \log _{a}\ell +\log _{a}\delta \right) \nonumber \\&\qquad \qquad \qquad -\Psi _{0}^{2}(\log _{a}\ell +\log _{a}\delta )+\mathcal {O}\left( \frac{\log ^{\rho +4}\ell }{\ell ^{\rho }}\right) , \end{aligned}$$

where $\gamma$ is the Euler–Mascheroni constant, and $\Psi _{0}(x)$ and $\Psi _{1}(x)$ are periodic functions (with period 1 and mean value 0), given by the Fourier expansions

15 $$\begin{aligned} \Psi _{0}(x)=-\frac{1}{\log a}\sum _{n\ne 0}\Gamma \left( -\frac{2n\pi i}{\log a} \right) e^{2n\pi ix},\end{aligned}$$

16 $$\begin{aligned} \Psi _{1}(x)=\frac{2}{\log ^{2}a}\sum _{n\ne 0}\Gamma '\left( -\frac{2n\pi i}{\log a} \right) e^{2n\pi ix}. \end{aligned}$$

### Proof

Parts of the proof of this theorem follow along the same lines as the proof of Lemma 2.5 in [7]. However, we include all steps of the proof for the sake of readability.

Without loss of generality, we can assume $r_{0}(1)/s_{0}(1)=1$, as otherwise $w_{\ell k}$ and $w_{\ell }$ are multiplied by a constant. Let $0\le k_1\le k_2\le k_3$ denote suitable constants.

For some $C>0$ such that there is no root of $s_0$ inside $\{z:|z|\le 1+2C\}$ and such that $(1+C)/a<1$, we have

$$\begin{aligned} |S_k(z)-(1-z)s_0(z)|=\mathcal {O}\bigl (\bigl ((1+C)/a\bigr )^k\bigr )<|(1-z)s_0(z)|\end{aligned}$$

for $|z|=1+C$ and $k\ge k_1$. By Rouché’s Theorem, we conclude that for $k\ge k_1$, $S_k(z)$ has exactly one simple root in the disk $\{z:|z|\le 1+C\}$.

Since ${{\mathrm{sgn}}}(S_k(1))={{\mathrm{sgn}}}(s_1(1))$ and ${{\mathrm{sgn}}}(S_k(1+1/k))=-{{\mathrm{sgn}}}(s_0(1))$ for $k\ge k_2$, the assumption $\delta >0$ implies that $S_k(z)$ has a real root $\zeta _k=1+\varepsilon _k$ with $0<\varepsilon _k<1/k$ for $k\ge k_2$. Inserting this in $S_k(1+\varepsilon _k)=0$ yields $\varepsilon _k=\mathcal {O}(1/a^k)$. Using $S_k(1+\varepsilon _k)=0$ again shows that

$$\begin{aligned} \varepsilon _k=\frac{\delta }{a^k}\bigl (1+\mathcal {O}(k/c^k)\bigr ), \end{aligned}$$

where $$\min \{a, |a_2|/a\}= a^\rho =: c>1$$.

Since $G_{k}$ and *G* are rational functions, $G_{k}$ and *G* can be continued analytically beyond their dominant singularities $\zeta _{k}$ and 1, respectively. We have $\lim _{k\rightarrow \infty }{{\mathrm{Res}}}_{z=\zeta _{k}}G_{k}(z)={{\mathrm{Res}}}_{z=1}G(z)=1$. Thus [11, Theorem 1] implies (12) and the limiting distribution.

The coefficients $w_{\ell k}$ and $w_{\ell }$ of $z^{\ell }$ in $G_{k}$ and *G*, respectively, coincide for $k\ge \ell$. Thus the support of $X_{\ell }$ is finite. Furthermore, the condition on $s_{0}$ implies that $w_{\ell }=1+\mathcal {O}(\kappa ^{\ell })$ for a constant $0\le \kappa <1$ by singularity analysis. Thus, the expectation is

17 $$\begin{aligned} \mathbb E X_{\ell }=\sum _{k\ge 0}k\mathbb P(X_{\ell }=k)=\sum _{k= 0}^{\ell }\Big (1-\frac{w_{\ell k}}{w_{\ell }}\Big )=\sum _{k=0}^{\ell }(1-w_{\ell k})+\mathcal {O}(\ell \kappa ^{\ell }). \end{aligned}$$

Using the residue theorem and the assumption $r_0(1)=s_0(1)$, we get

$$\begin{aligned} w_{\ell k}&={{\mathrm{Res}}}_{z=0}\frac{R_k(z)}{z^{\ell +1}S_k(z)}\\&= \frac{1}{2\pi i}\oint _{|z|=1+C/2} \frac{R_k(z)}{z^{\ell +1}S_k(z)} - {{\mathrm{Res}}}_{z=\zeta _k}\frac{R_k(z)}{z^{\ell +1}S_k(z)}\\&= -\frac{R_k(\zeta _k)}{S'_k(\zeta _k)}\zeta _k^{-(\ell +1)} + \mathcal {O}((1+C/2)^{-\ell })\\&= \exp (-\ell \delta /a^k)\bigl (1+\mathcal {O}(k/a^k)+\mathcal {O}(\ell k/(a^kc^k))\bigr )+ \mathcal {O}((1+C/2)^{-\ell }) \end{aligned}$$

for $$k_3\le k\le n$$.

Replacing $w_{\ell k}$ with $\exp (-\ell \delta /a^{k})$ yields the error terms

18 $$\begin{aligned} |w_{\ell k}-\exp (-\ell \delta /a^{k})|= {\left\{ \begin{array}{ll} \mathcal {O}(\ell ^{-2})&{}\text {for }0\le k\le \log _{a}(\ell \delta /(4 \log \ell )),\\ \mathcal {O}(\log _{a}^{\rho +2}\ell /\ell ^{\rho })&{}\text {for }\log _{a}( \ell \delta /(4\log \ell ))\le k\le 5\log _{a}\ell ,\\ \mathcal {O}(\ell ^{-3})&{}\text {for }5\log _{a}\ell \le k\le \ell \end{array}\right. } \end{aligned}$$

where we used $w_{\ell k} \le w_{\ell k_3}$ for $k\le k_3$. As $1-\exp (-\ell \delta /a^{k})$ is exponentially small for $k>\ell$, we obtain

19 $$\begin{aligned} \sum _{k=0}^\ell (1-w_{\ell k})=\sum _{k=0}^\infty \bigl (1-\exp (-\ell \delta / a^k)\bigr ) + O\left( \frac{\log ^{\rho +3} \ell }{\ell ^\rho }\right) . \end{aligned}$$

Thus, (13) follows from (17), (19) and the well known fact (see e.g. [2]) that

$$\begin{aligned} \sum _{k\ge 0}\big (1-e^{- x/a^k}\big ) =\log _ax+\frac{\gamma }{\log a}+\frac{1}{2}+\Psi _{0}(\log _ax)+ \mathcal {O}(x^{-1}) \end{aligned}$$

with the periodic function $\Psi _{0}(x)$ given in (15).

The second moment is

20 $$\begin{aligned} \begin{aligned} \mathbb E X_{\ell }^{2}&=\sum _{k\ge 0}k^{2}\mathbb P(X_{\ell }=k)=\sum _{k=0}^{\ell }(2k+1)\Big (1-\frac{w_{\ell k}}{w_{\ell }}\Big )\\&=\sum _{k=0}^{\ell }(2k+1)(1-w_{\ell k})+\mathcal {O}(\ell ^{2}\kappa ^{\ell }). \end{aligned} \end{aligned}$$

As $\sum _{k=0}^{\ell }(1-w_{\ell k})$ has already been computed for the expectation, we are left with $\sum _{k=0}^{\ell }k(1-w_{\ell k})$. We use (18) to obtain

21 $$\begin{aligned} \sum _{k=0}^{\ell }k(1-w_{\ell k})=\sum _{k\ge 0}k(1-\exp (-\ell \delta /a^{k}))+\mathcal {O}\Big (\frac{\log ^{\rho +4}\ell }{\ell ^{\rho }}\Big ). \end{aligned}$$

The Mellin transform (see [2]) of the harmonic sum $F(x)=\sum _{k\ge 0}k(1-\exp (-x/a^{k}))$ is

$$\begin{aligned} F^{*}(s)=\frac{-a^{s}}{(1-a^{s})^{2}}\Gamma (s) \end{aligned}$$

for $-1<\mathfrak {R}s<0$. The singular expansion of this Mellin transform at $\mathfrak {R}s=0$ is

$$\begin{aligned} F^{*}(s)&\asymp -\frac{1}{\log ^{2}a}s^{-3}+\frac{\gamma }{\log ^{2}a}s^{-2}+\left( \frac{1}{12}-\frac{1}{2\log ^{2}a}\left( \gamma ^{2}+\frac{\pi ^{2}}{6}\right) \right) s^{-1}\\&\quad - \sum _{n\ne 0}\frac{\Gamma (-\chi _{n})}{\log ^{2}a} (s+\chi _{n})^{-2}\\&\quad -\sum _{n\ne 0}\frac{\Gamma '(-\chi _{n})}{\log ^{2}a} (s+\chi _{n})^{-1} \end{aligned}$$

for $$\chi _{n}=\frac{2\pi i n}{\log a}$$. Thus,

$$\begin{aligned} F(x)&=\frac{1}{2}\log _{a}^{2}x+\frac{\gamma }{\log a}\log _{a}x-\frac{1}{12}+\frac{1}{2\log ^{2}a}\left( \gamma ^{2}+\frac{\pi ^{2}}{6}\right) \\&\quad -\frac{\log _{a}x}{\log a}\sum _{n\ne 0}\Gamma (-\chi _{n})\exp (2\pi in\log _{a} x)\\&\quad +\frac{1}{\log ^{2}a}\sum _{n\ne 0}\Gamma '(-\chi _{n})\exp (2\pi in\log _{a} x)+\mathcal {O}(x^{-1}). \end{aligned}$$

Thus, $\mathbb VX_{\ell }=\mathbb EX_{\ell }^{2}-(\mathbb EX_{\ell })^{2}$, (20), (17), (13) and (21) give the variance as stated in (14). $\square$

### Theorem 4

Let $q\ge 2$ be even. Then the expected number of iterations when adding two SSDE of length $\ell$ with von Neumann’s algorithm is

22 $$\begin{aligned} \log _{q}\ell +\log _{q}\delta +\frac{\gamma }{\log q}+\frac{5}{2}+\Psi _{0}(\log _{q}\ell +\log _{q}\delta )+\mathcal {O}(\ell ^{-1}\log ^{4}\ell ) \end{aligned}$$

where

$$\begin{aligned} \delta =\frac{(q - 1)(4q^{10} + 10q^9 + 18q^8 - 4q^7 - 10q^6 + 7q^5 + 44q^4 - 29q^3 - 8q^2 - 20q + 16)}{4q^{3}(q + 1)^2(4q^7 - q^5 - 6q^4 + 8q^3 + 2q - 4)}, \end{aligned}$$

$\Psi _{0}(x)$ is a 1-periodic function with mean 0 given by the Fourier expansion

23 $$\begin{aligned} \Psi _{0}(x)=-\frac{1}{\log q}\sum _{k\ne 0}\Gamma \left( -\frac{2k\pi i}{\log q}\right) e^{2k\pi ix}. \end{aligned}$$

The variance of the number of iterations is

24 $$\begin{aligned} \frac{\pi ^{2}}{6\log ^{2}q}+\frac{1}{12}+\Psi _{1}(\log _{q}\ell +\log _{q}\delta )-\frac{2\gamma }{\log q}\Psi _{0}(\log _{q}\ell +\log _{q}\delta )-\Psi _{0}^{2}(\log _{q}\ell +\log _{q}\delta )+\mathcal {O}(\ell ^{-1}\log ^{5}\ell ) \end{aligned}$$

where $\Psi _{1}$ is a 1-periodic function with mean 0 given by the Fourier expansion

25 $$\begin{aligned} \Psi _{1}(x)=\frac{2}{\log ^{2}q }\sum _{k\ne 0}\Gamma '\left( -\frac{2k\pi i}{\log q}\right) e^{2k\pi ix}. \end{aligned}$$

The asymptotic formula

$$\begin{aligned} \mathbb P_{\ell }(t(\varvec{x},\varvec{y})\le k)=\exp (-\delta \ell /q^{k})(1+o(1)) \end{aligned}$$

holds as $\ell \rightarrow \infty$ for $k=\log _{q}\ell +\mathcal {O}(1)$. The random variable $t(\varvec{X},\varvec{Y})-\log _{q}\ell$ converges weakly to a double exponential random variable if $\ell$ runs through a subset of the positive integers such that the fractional part $\{\log _{q}\ell \}$ converges.

### Remark 7.1

A similar result for $q\ge 4$ was obtained in [7] using the same probabilistic model as in Remark 5.5. This changes the main term of the expected value. In [7], the logarithm of the main term was taken to the base $\alpha ^{-1}$ with $\alpha =q^{-1}-q^{-4}+\mathcal {O}(q^{-5})$. In contrast, we here obtain the logarithm of the main term in (22) to the base *q*, which is a more natural constant appearing in this context.

For $q=2$, this result is contained in [7].

### Proof

Let $\mathbb P_{\ell }$ be the (exact) equidistribution of all SSDE of length $\ell$. For $k>\ell +2$, we know that $\mathbb P_{\ell }(t(\varvec{X}, \varvec{Y})\le k)=1$ because an input sequence of length $\ell$ traverses at most $\ell$ solid edges in the automaton in Fig. 8.

If we use the approximate equidistribution $W_{\ell }=(1+\mathcal {O}(\xi ^{\ell }))\mathbb P_{\ell }$ of all SSDE of length $\ell$, an exponentially small error term is introduced. Because of the finite support, this error term does not change the main term of the expectation, the variance and the distribution function. Thus, also the limiting distribution remains the same.

We will use Theorem 3 with the generating function

$$\begin{aligned} G_{k}(z)=\sum _{\ell \ge 0}w_{\ell k}z^{\ell } \end{aligned}$$

for $w_{\ell k}=W_{\ell }(t(\varvec{x},\varvec{y})-2\le k)$, $k\ge 0$. To construct this generating function, we use the same techniques as in [7].

The generating function $G_{k}(z)$ counts the weighted number of paths in the automaton $\mathcal {N}_{\text {SSDE}}$ of the pattern $\ldots \mathcal B^{+}\mathcal R^{\{1, k\}}\mathcal B^{+}\mathcal R^{\{1, k\}}\ldots$ where $\mathcal B^{+}$ is an arbitrary non-empty sequence of dotted transitions and $\mathcal R^{\{1, k\}}$ is a non-empty sequence of solid transitions of length at most *k*. The first transition can be a dotted or a solid transition. We stop with either arbitrarily many dotted transitions or at most *k* solid transitions, where we have to take into account the special situation in states 2 or 7 in the automaton in Fig. 8 (see also Remark 6.1): Because of the solid transition starting in 2 and 7 with label 0, we are not allowed to stop with *k* solid transitions in state 2 or 7 but only with at most $k-1$ ones.

To find the generating functions for $\mathcal B^{+}$ and $\mathcal R^{\{1, k\}}$, we use the transition matrices for the dotted and the solid parts of the automaton $\mathcal {N}_{\text {SSDE}}$.

Let $q\ge 6$. The transition matrix *R* for the solid transitions of automaton $\mathcal {N}_{\text {SSDE}}$ is a $12\times 12$ matrix given in Table 9 in the appendix (using Lemma 5.2). The transition matrix *B* for the dotted transitions of automaton $\mathcal {N}_{\text {SSDE}}$ is given in Table 10 (using Lemma 5.2). The order of the states is given in (11) and also in Table 8 in the appendix.

The (matrix) generating function for arbitrary non-empty dotted paths $\mathcal B^{+}$ is

$$\begin{aligned} B^{+}(z)=(I-zB)^{-1}-I. \end{aligned}$$

The entry (*i*, *j*) of this matrix is the generating function of non-empty dotted paths of arbitrary length starting in state *i* and leading to state *j*. For arbitrary non-empty solid paths, the (matrix) generating function is

$$\begin{aligned} R^{+}(z)=(I-zR)^{-1}-I. \end{aligned}$$

To obtain the (matrix) generating function $R^{\{1, k\}}$ for non-empty solid paths $\mathcal R^{\{1, k\}}$ of length at most *k*, we have to restrict each entry of $R^{+}$ corresponding to an infinite geometric series to a finite geometric series.^3^ We will illustrate this procedure on

26 $$\begin{aligned} \frac{- q^{4} z + 10 q^{3} z - 3 q^{2} z^{2} - 24 q^{2} z + 10 q z^{2} + 8 z^{2}}{-8 q^{4} + 8 q^{3} z - 8 q^{2} z + 8 q z^{2}}, \end{aligned}$$

the entry at position (5, 1) of $R^{+}$. The partial fraction decomposition of (26) with respect to *z* is

$$\begin{aligned} -\frac{(3q+2)(q-4)}{8 q}+ \frac{(q-4)(q+4) }{4 (q + 1)}\cdot \frac{1}{ 1 + z/q^{2}} +\frac{(q-1)(q-2)(q-4)}{8 q (q + 1)}\cdot \frac{1}{1 - z/q}. \end{aligned}$$

By truncating the infinite geometric sum $(1-z)^{-1}$ after $k+1$ summands, i.e., by replacing it with $(1-z^{k+1})(1-z)^{-1}$, we obtain

$$\begin{aligned}&-\frac{(3q+2)(q-4)}{8 q}+ \frac{(q-4)(q+4)}{4 (q + 1)}\cdot \frac{1-(-z/q^{2})^{k+1}}{ 1 + z/q^{2}}\\&+ \frac{(q-1)(q-2)(q-4)}{8 q (q + 1)}\cdot \frac{1-(z/q)^{k+1}}{1 - z/q}. \end{aligned}$$

Let

$$\begin{aligned} M_{k}(z)=\begin{pmatrix} 0&{}B^{+}(z)\\ R^{\{1, k\}}(z)&{}0 \end{pmatrix} \end{aligned}$$

be the block matrix of total size $24\times 24$. Then, the (matrix) generating function of non-empty paths $\ldots \mathcal B^{+}\mathcal R^{\{1, k\}}\mathcal B^{+}\mathcal R^{\{1, k\}}\ldots$ is

$$\begin{aligned} (I-M_{k}(z))^{-1}-I. \end{aligned}$$

To take into account the initial states and the exit weights in the automaton $\mathcal {N}_{\text {SSDE}}$, we define the initial vector

$$\begin{aligned} u=(0, 0, 0, 0, 0, 0, 0, 0, 1, 0, 0, 0;0, 0, 0, 0, 0, 0, 0, 0, 1, 0, 0, 0). \end{aligned}$$

Using the exit weights in Table 8 in the appendix, we further define the exit vector

$$\begin{aligned}&v^{\top }=\Big (\frac{q+1}{q+2}\Big )^{2} (4, 1, 2, 0, 1, 0, 2, 2, 1, 1, 4, 2; 4, 1, 2, 2, 1, 1, 2, 2, 1, 1, 4, 2)^{\top }\\&\qquad \qquad +\Big (\frac{q+1}{q+2}\Big )^{2}M_{k-1}(z)( 0,0,0,2,0,1,0,0,0,0,0,0; 0,0,0,0,0,0,0,0,0,0,0,0)^{\top } \end{aligned}$$

taking into account the special situation with states 2 and 7 in the automaton in Fig. 8.

Then, the generating function is

$$\begin{aligned} G_{k}(z)=u((I-M_{k}(z))^{-1}-I)v+\Big (\frac{q+1}{q+2}\Big )^{2} \end{aligned}$$

where we add the exit weight of state (1, (0, 0)) because the empty word was not counted until now. The result is

27 $$\begin{aligned} G_{k}(z)=\frac{r_{0}(z)+\Big (\frac{z}{q}\Big )^{k}r_{1}\Big (z, \Big (\frac{z}{q}\Big )^{k}, \Big ({-}\frac{z}{q^{2}}\Big )^{k}\Big )}{(1-z)s_{0}(z)+\Big (\frac{z}{q}\Big )^{k}s_{1}(z)+\Big ({-}\frac{z}{q^{2}}\Big )^{k}s_{2}\Big (z, \Big (\frac{z}{q}\Big )^{k}, \Big ({-}\frac{z}{q^{2}}\Big )^{k}\Big )} \end{aligned}$$

with

$$\begin{aligned} r_{0}(z)&=4 q^{7} (q + 1)^{3} (4 z^{2} - 3 q^{2} z- q^{3}) ( 2 q z^{4}- 4 z^{4}+ 8 q^{3} z^{2}- q^{5} z^{2} - 6 q^{4} z^{2}+4 q^{7} ),\\ s_{0}(z)&=4 q^{7} (q + 1) (q + 2)^{2} (z+q) (z- q^{2}) (2 q z^{4}- 4 z^{4}+8q^{3} z^{2} - q^{5} z^{2} - 6 q^{4} z^{2} +4 q^{7} ),\\ s_{1}(z)&=- (q + z) z^2 (q + 2)^2 q^4 (4q^{12} + 6q^{11}z + 2q^{10}z^2 - 4q^{11} - 24q^{10}z\\&\quad - 8q^9z^2 + 24q^{10} + 26q^9z + 4q^8z^2 + 5q^7z^3 - 7q^6z^4 - 48q^9 \\&\quad - 20q^8z + 18q^7z^2 - 9q^6z^3 + 34q^5z^4 + 5q^4z^5 + 32q^8 + 36q^6z^2 \\&\quad - 32q^5z^3 - 59q^4z^4 - 25q^3z^5 - 112q^5z^2 + 84q^4z^3 + 40q^3z^4 \\&\quad + 44q^2z^5 + 64q^4z^2 - 48q^3z^3 + 4q^2z^4 - 36qz^5 - 16qz^4 + 16z^5) \end{aligned}$$

and some polynomials $r_{1}$ and $s_{2}$ in *z*, $(z/q)^{k}$ and $(-z/q^{2})^{k}$ with coefficients in $\mathbb Q[q]$. The polynomial $s_{0}$ does not have any zeros in the closed unit disc. We have $r_{0}(1)\ne 0$ and $\delta >0$.

For $q\le 4$, the construction of the generating function $G_{k}(z)$ is the same, only the matrices *R* and *B* and the vectors *u* and *v* are slightly different. Nevertheless, (27) including the definitions of all the occurring polynomials is still valid.

By Theorem 3, we obtain the expectation, the variance, the distribution function and the limiting distribution of the non-negative truncation of $t(\varvec{x},\varvec{y})-2$. From (18) and the monotonicity of $w_{\ell k}$, we know that $w_{\ell -2}=w_{\ell -1}=\mathcal {O}(\ell ^{-2})$. Therefore, the results transfer to the random variable $t(\varvec{x}, \varvec{y})$ as stated in the theorem. $\square$

## Appendix A: Transition matrices

In this appendix in Tables 6, 7, 8, 9 and 10, we show the transition matrices and exit weights which are used in Sects. 5 and 7.

**Table 6:** Transition matrix of $\mathcal {S}_{(q,d)}$ in Sect. 5.1 multiplied with $2q^{2}$

$(-1,(0,0))$	(0, (0, 0))	(1, (0, 0))
$(d - 1)(d - 2)y$	$-2d^2 - 2dq + q^2 + 6d + 3q - 4$	$(d + q - 1)(d + q - 2)x$
$(d - 1)dy$	$-2d^2 - 2dq + q^2 + 2d +q$	$(d + q)(d + q - 1)x$
$(d + 1)d y$	$-2d^2 - 2dq + q^2 - 2d -q$	$(d + q + 1)(d + q)x$

The order of the states is given in the first line

**Table 7:** Transition matrix of $\mathcal {S}_{\text {SSDE}}$ for $q\ge 8$ in Sect. 5.2 multiplied with $8q^{2}$

$$ 6q^2 - 12q + 8 $$	4	$$ 4(q-2)y $$	8	$$ 4q-8 $$	$$ 4q-8 $$	8	$$ 4(q-2)x $$	$$ (q-2)(q-4)y $$	$$ (q-2)(q-4)x $$	2*y*	2*x*	$$ 4q-8 $$	$$ 4q-8$$
$$8q^2 $$	0	0	0	0	0	0	0	0	0	0	0	0	0
$$6(q-2)q $$	0	4*qy*	0	0	4*q*	0	0	$$ 2(q-2)qy $$	0	0	0	8*q*	0
$$2(qy + 2q - 2y)q $$	0	$$ 4qy^2 $$	0	0	4*qy*	0	0	$$ 2(q-2)qy^2 $$	0	0	0	0	0
$$2(3q - 2)q $$	0	$$ 4(q-2)y $$	8	0	$$ 4q-8 $$	8	0	$$ 2(q-2)(q - 4)y $$	0	0	0	$$ 8q-16 $$	0
$$2(3q-2)q $$	0	0	8	$$ 4q-8 $$	0	8	$$ 4(q-2)x $$	0	$$ 2(q-2)(q-4)x $$	0	0	0	$$ 8q-16$$
$$2(qx + 2q - 2x)q $$	0	0	0	4*qx*	0	0	$$ 4qx^2 $$	0	$$ 2(q-2)qx^2 $$	0	0	0	0
$$6(q-2)q $$	0	0	0	4*q*	0	0	4*qx*	0	$$ 2(q - 2)qx $$	0	0	0	8*q*
$$6(q-2)q $$	4	4*qy*	8	$$ 4q-16 $$	4*q*	8	$$ 4(q-4)x $$	$$ (q-2)qy $$	$$ (q-4)(q-6)x $$	2*y*	2*x*	4*q*	$$ 4q-16$$
$$6(q-2)q $$	4	$$ 4(q-4)y $$	8	4*q*	$$ 4q-16 $$	8	4*qx*	$$ (q-4)(q-6)y $$	$$ (q-2)qx $$	2*y*	2*x*	$$ 4q - 16 $$	4*q*
$$4(q-2)q $$	0	0	0	0	0	0	0	$$ 4(q-2)qy $$	0	0	0	16*q*	0
$$4(q-2)q $$	0	0	0	0	0	0	0	0	$$ 4(q-2)qx $$	0	0	0	16*q*
$$(3qy + 3q - 6y - 2)q $$	4	$$ 4qy^2 $$	0	$$ 4q-8 $$	4*qy*	0	$$ 4(q-2)x $$	$$ (q-2)qy^2 $$	$$ (q - 2)(q-4)x $$	2*y*	2*x*	0	0
$$(3qx + 3q - 6x - 2)q $$	4	$$ 4(q-2)y $$	0	4*qx*	$$ 4q - 8 $$	0	$$ 4qx^2 $$	$$ (q - 2)(q - 4)y $$	$$ (q - 2)qx^2 $$	2*y*	2*x*	0	0

The order of the states is $\{(0,(0, 0))\}$, $\{(0,(-1, 1)), (0,(1,-1))\}$, $\{(-1,(-1, 0)), (-1,(0, -1))\}$, $\{(-q/2,(-1, 0)), (-q/2,(0, -1))\}$, $\{(0,(-1, 0)), (0,(0, -1))\}$, $\{(0,(0, 1)),(0,(1, 0))\}$, $\{(q/2,(0, 1)), (q/2,(1, 0))\}$, $\{(1,(0, 1)), (1,(1, 0))\}$, $\{(-1,(0, 0))\}$, $\{(1,(0, 0))\}$, $\{(-1,(-1,-1))\}$, $\{(1,(1, 1))\}$, $\{(-q/2,(0, 0))\}$, $\{(q/2,(0, 0))\}$

**Table 8:** Exit weights of $\mathcal {N}_{\text {SSDE}}$ in Sect. 7 multiplied with $\big (\frac{q+2}{q+1}\big )^{2}$

$(1,(-1, 1))$	(4, (0, 0))	(5, (0, 1))	(2, (0, 1))	(5, (0, 0))	(2, (0, 0))	$(1,(-1, 0))$	(3, (0, 1))	(1, (0, 0))	(3, (0, 0))	(4, (1, 1))	(4, (0, 1))
4	1	2	2	1	1	2	2	1	1	4	2

As discussed in (11), the states of $\mathcal {N}_{\text {SSDE}}$ are equivalence classes of states. For brevity, we list one representative for each state of $\mathcal {N}_{\text {SSDE}}$ to give the order of the states

**Table 9:** Transition matrix *R* for the solid transitions in $\mathcal {N}_{\text {SSDE}}$ for $q\ge 6$ in Sect. 7 multiplied with $8q^{2}$

0	0	0	0	0	0	0	0	0	0	0	0
0	0	0	0	0	0	0	0	0	0	0	0
0	0	0	0	0	0	0	0	0	0	0	0
0	0	0	0	0	0	0	0	$4q(q-2)$	8*q*	0	0
0	0	0	0	0	0	0	0	0	0	0	0
4	$$ (q-4)(q-6) $$	0	8	0	$$ 4(q-4) $$	$$ 4(q-4) $$	8	$$ 3q(q-2)$$	4*q*	4	$$ 4 (q-4)$$
0	0	0	0	0	0	0	0	0	0	0	0
0	$$ 2(q-2)(q-4) $$	0	8	0	$$ 8 (q-2) $$	$$ 4 (q-2) $$	8	$$ 2q(q-2) $$	0	0	$$ 4 (q-2)$$
0	0	0	0	0	0	0	0	0	0	0	0
0	$$ (q-2)(q-4) $$	0	8	0	$$ 4 (q-2) $$	$$ 4 (q-2) $$	8	$$ (3q-4)(q - 2)$$	$$ 4 (q-2) $$	0	$$ 4 (q - 2)$$
0	0	0	0	0	0	0	0	0	0	0	0
0	0	0	0	0	0	0	0	0	0	0	0

The order of the states is the same as in Table 8

**Table 10:** Transition matrix *B* for the dotted transitions in $\mathcal {N}_{\text {SSDE}}$ for $q\ge 6$ in Sect. 7 multiplied with $8q^{2}$

0	0	0	0	0	0	0	0	$8 q^{2}$	0	0	0
4	$$ 2 (q - 4)^{2} $$	0	16	0	$$ 8 (q-3) $$	$$ 8 (q-3) $$	16	$$ 2 (3 q-2) (q-2) $$	$$ 8 (q-1) $$	4	$$ 8(q-3)$$
0	$$ 2 (q-2) (q-4) $$	0	8	0	$$ 8(q-2) $$	$$ 4 (q-2) $$	8	$$ 2 q(3 q-2) $$	0	0	$$ 4(q-2)$$
0	$2 (q-2) (q-4)$	0	8	0	$8(q-2)$	$4 (q-2)$	8	$2q(q - 2)$	0	0	$4(q-2)$
4	$$ 2 (q-2)(q-4) $$	0	8	0	$$ 4(q-2) $$	$$ 8 (q-2) $$	8	$$ 6q^{2} - 12q + 8 $$	$$ 4 (q-2) $$	4	$$ 8(q-2)$$
0	$$ (q-2) (q-4) $$	0	8	0	$$ 4(q-2) $$	$$ 4 (q-2) $$	8	$$ (3 q-4)(q-2) $$	$$ 4( q-2) $$	0	$$ 4(q-2)$$
0	$$ 2 (q-2) (q-4) $$	16	0	$$ 8(q-2) $$	0	$$ 4 (q-2) $$	0	$$ 2q(3q-2) $$	0	0	$$ 4(q-2)$$
0	0	0	0	0	0	0	0	$4q^{2}$	0	0	0
4	$2 (q-2)(q-4)$	16	0	$8(q-2)$	0	$8 (q-2)$	0	$6q^{2} -12 q + 8$	0	4	$8(q-2)$
4	$$ (q-2) (q-4) $$	0	0	0	0	$$ 4(q-2) $$	0	$$ q(3 q - 2) $$	0	4	$$ 4(q-2)$$
0	$4 (q-2) (q-4)$	0	0	0	$16(q-2)$	0	0	$4 q(q-2)$	16*q*	0	0
0	$2(q-2)(q-4)$	0	8	0	$8(q-2)$	$4(q-2)$	8*z*	$6(q-2)q$	8*q*	0	$4(q-2)$

The order of the states is the same as in Table 8

## Footnotes

1 This can also be seen as the composition of a transducer performing digitwise addition (without considering any carries) and the Cartesian product of $\mathcal {A}$ with itself. This corresponds to the SageMath methods transducers.add and Transducer.cartesian_product, respectively. The composition can be computed by the SageMath method Transducer.composition.

2 For the actual computation, it is more efficient to already simplify $\mathcal {A}^{2}$ by the SageMath method FiniteStateMachine.markov_chain_simplification, such that it only has 6 states.

3 It is also possible to use $R^{\{1,k\}}(z)=zR+\cdots +z^{k}R^{k}=(I-z^{k+1}R^{k+1})(I-zR)^{-1}-I$. However, this involves a power of the *symbolic* matrix *R* with the *symbolic* exponent *k*. This would require a full symbolic eigenvalue decomposition of *R*. The approach chosen here avoids this by introducing the length restriction on each entry individually.
